# Development of Novel Erythromycin Derivatives with Inhibitory Activity against Proliferation of Tumor Cells

**DOI:** 10.1371/journal.pone.0159503

**Published:** 2016-07-22

**Authors:** Lan Wu, Kai Bao, Rui Song, Defa Wang, Lei Zhang, Weiyun Wang, Weige Zhang, Wen Bin

**Affiliations:** 1 Department of Geratology, The First Affiliated Hospital of Chinese Medical University, Shenyang, China; 2 State Key Laboratory of Bioactive Substance and Function of Natural Medicines, Institute of Materia Medica, Chinese Academy of Medical Sciences and Peking Union Medical College, Beijing, China; 3 Key Laboratory of Structure-Based Drug Design and Discovery Ministry of Education, Shenyang Pharmaceutical University, Shenyang, China; 4 School of Life Sciences and Biopharmaceutics, Shenyang Pharmaceutical University, Shenyang, China; National Cancer Institute at Frederick, UNITED STATES

## Abstract

In our continuing structure-activity relationship study of a new class of erythromycin A (EM-A) derivatives with antiproliferative activity, a new series of de(N-methyl) EM-A dimers jointed by a four-atom linker, -CH_2_CH = CHCH_2_-, were prepared and their antiproliferative activity against three human tumor cell lines was evaluated by MTT assay. The most active EM-A dimer, compound **1b**, that carrying C6 methoxyl groups was further investigated and showed potent antiproliferative activity in six other human tumor cell lines. Flow cytometry analysis of **1b** treated HeLa and MCF-7 cells indicated that the four-atom EM-A dimers induced the SubG_1_ phase cell cycle arrest and cell apoptosis, in time- and dose-dependent manners. Further experiments including morphologic observation, DNA agarose gel electrophoresis, mitochondrial potential alternation and western blot analysis revealed that the antiproliferative mechanism may involve the induction of apoptosis in activating the mitochondrial pathway, and regulation of apoptotic proteins.

## Introduction

Over the past decade, significant progress has been achieved in the development of novel erythromycin A (EM-A) derivatives with improved antimicrobial activity [1–3]. In addition to the antibacterial effect, more attentions have been attracted in the study of other bioactivities of EM-A derivatives including gastrointestinal prokinetic activity [4,5], anti-inflammatory activity [6–9] and antiproliferative activity [10,11], as new therapeutic potentials.

Increasing evidence demonstrates that EM-A and its derivatives ameliorate antitumor potentials *via* mechanisms independent of its antibacterial activity. The antitumor mechanisms may involve reversal of antitumor drug resistance [12,13], immunoregulation [14], inhibition of tumor angiogenesis [15,16], modulation of human ether-a-go-go-related gene (HERG) potassium channels [17], and inhibition of histone deacetylase (HDAC) [18,19]. In previous studies, we have reported the *in vitro* antiproliferative activity of a new class of dimers of de(*N*-methyl) EM-A derivatives, linked by a -COCH_2_- or -CH_2_CH_2_- linker [11]. Flow cytometry studies revealed that the dimers’ antiproliferative mechanism involves inducing cell-cycle arrest in the G_0_/G_1_ phase.

Although there are numerous reports of EM-A and its derivatives having antitumor activity, the exact mechanisms remain elusive, and only a little attention has been paid to the structure-activity relationship (SAR) study for the treatment of tumor diseases. This paper presents our continuing SAR research of a new class of EM-A derivatives with antiproliferative activity. We evaluated the *in vitro* antiproliferative effects of a series of dimers of EM-A derivatives against different human tumor cell lines by 3-(4,5-dimethylthiazol-2-yl)-2,5-diphenyltetrazolium bromide (MTT) assay. Preliminary mechanism study by using flow cytometric analysis, DNA agarose gel electrophoresis, western blot analysis etc. confirmed that the antiproliferative activity of the EM-A dimers may involve the induction of cell apoptosis.

## Results and Discussion

### Chemistry

**Fig 1:**
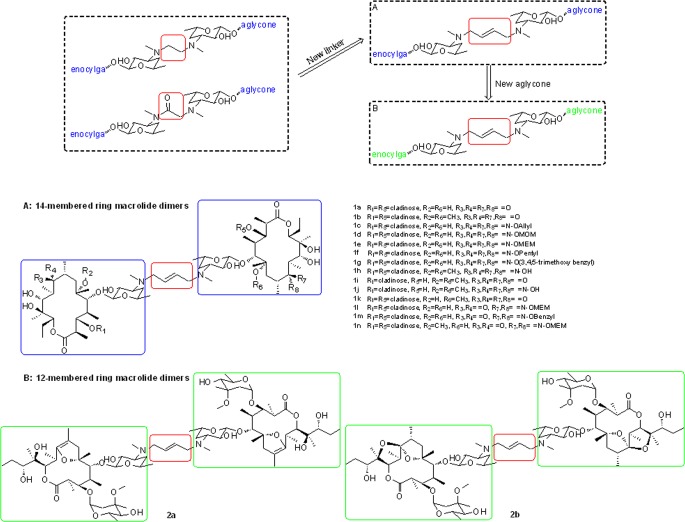
Strategy for the design of dimers of EM-A derivatives **1a-2b**.

For EM-A dimers, our previous SAR study indicates that the presence of a two-atom linker, -COCH_2_- or -CH_2_CH_2_-, between the two de(N-methyl) EM-A units is essential for antiproliferative activity. To investigate the effects of the length and structure variations of the linker on antiproliferative activity, a four-atom linker, -CH_2_CH = CHCH_2_-, was introduced to join the two de(N-methyl) EM-A units (Fig 1A). Modifications at the C3, C6 and C9 positions on the de(N-methyl) EM-A units have significant influences on antiproliferative activity. Accordingly, we designed EM-A homodimer **1a**, **1b** that carrying an methoxy substitution at C6 positions, **1c-1g** that has an O-alkyloxime group at C9 positions, and **1h** that carrying an O-methyl substitution at C6 positions and unsubstituted oxime groups at C9 positions; EM-A heterodimers **1i** and **1j** that losing a cladinose at C3 position of one unit, and **1k** -**1n** with modifications at C6 and C9 position of one unit.

12-membered EM-A derivatives were reported to show potent in *vitro*/*vivo* anti-inflammatory and immunomodulatory activity [20]. To assess the importance of the aglycone size and further expand the diversity of the EM-A dimers, homodimers **2a** and **2b** with 12-membered aglycones were designed (Fig 1B).

According to the synthetic strategy in our previous work, all of the target compounds were synthesized through alkylation of the de(N-methyl) EM-A derivatives **3a–3m** by using (*E*) 1,4-dibromo-2-buten in the presence of N, N-diisopropylethylamine in anhydrous dichloromethane (19% to 85% yields). (S1 Fig). All the final dimers produced in this study were fully characterized by MS, ^1^H NMR, ^13^C NMR and element analysis as described in the experimental section of the Supporting Information.

### Bioassay

#### Antiproliferative activity assay

*In vitro* antiproliferative activity against three human tumor cell lines, including gastric adenocarcinoma (SGC-7901), oral carcinoma (KB) and fibrosarcoma (HT-1080), was evaluated using the MTT assay with *cis*-platinum (Cpt) as the positive control. The drug concentrations required to inhibit cell growth by 50% (IC_50_) following incubation in culture medium for 24 h are displayed in Table 1. The IC_50_ values obtained for Cpt in this assay are 4.9, 4.7 and 19.7 μM for SGC-7901, KB and HT-1080 cell lines, respectively.

**Table 1:** *In vitro* inhibitory effects of compounds 1a-2b against the proliferation of three human cancer cell lines.

Compd.	IC_50_ μM ^a^			
	SGC-7901	KB	HT-1080	L929
**1a**	13.9±1.5	9.6±1.1	10.3±0.9	>100
**1b**	2.5±0.3	0.3±0.1	1.3±0.2	72.1±1.6
**1c**	27.3±1.8	4.7±0.5	6.8±0.7	>100
**1d**	0.5±0.1	4.6±0.5	5.1±0.4	>100
**1e**	12.3±1.1	7.8±0.8	6.9±0.5	>100
**1f**	6.6±0.5	2.7±0.2	1.5±0.2	>100
**1g**	24.5±2.2	21.6±1.8	21.1±1.9	>100
**1h**	3.7±0.3	2.8±0.2	2.6±0.2	>100
**1i**	27.1±2.2	3.1±0.3	11.9±0.8	>100
**1j**	30.6±2.8	26.4±2.4	23.7±2.2	>100
**1k**	5.0±0.4	5.5±0.4	2.0±0.2	>100
**1l**	15.8±1.2	7.6±0.6	7.0±0.6	>100
**1m**	28.5±2.5	8.3±0.6	5.7±0.4	>100
**1n**	6.9±0.4	4.4±0.3	3.6±0.2	>100
**2a**	5.7±0.5	>100	17.1±1.4	>100
**2b**	29.9±1.4	>100	>100	>100
**clarithromycin**	>100	>100	>100	>100
**Cpt**	4.9±0.4	4.7±0.3	19.7±1.5	18.5

^a^ IC_50_, expressed as the concentration of drug inhibiting cell growth by 50% after 24 h of drug exposure. All of the values were mean±SD of 3 separate experiments.

As shown in Table 1, transformation of the two-atom linker into four-atom linker did not lead to loss of biological activity but to an increase in potency for compounds with 14-membered lactone rings. For example, compound **1a** showed IC_50_ values of 13.9, 9.6 and 10.3μM against the SGC-7901, KB and HT-1080 cell lines, compared with 26.5, >100 and >100 for compound with -COCH_2_- linker, and 18.3, 37.3 and >100 for compound with -CH_2_CH_2_- linker [11]. On the other hand, contraction and dimerization of 14-membered lactone rings (compound **2a** and **2b**) resulted in a marked decrease in the activity of compound **2a** against KB cell lines and **2b** against KB and HT-1080 cell lines, which indicated a certain selectivity of the dimeric 12-membered aglycones for antiproliferative activity. Moreover, none of the dimeric compounds show any discernible toxicity against normal mouse fibroblast cells (L929) at drug concentrations in excess of 100 μM, except compound **1b** (IC_50_ 72.1 μM).

Compounds **1b**, **1h** and **1k** with C6 methoxy groups was found to have potent activity against all three cell lines, indicating that the structural modifications at C6 position had a beneficial effect on antiproliferative activity. Generally, the 9-oxime dimers **1c**-**1g, 1l** and **1m**, that have O-alkyloxime groups were found to be similar potent as **1a**, suggesting that conversion of at C9 carbonyl into O-alkyloxime groups had limited influence on the growth inhibition of the three cell lines. Comparing the IC_50_ values of compounds **1b** and **1h** with their decladinose relatives **1i** and **1j**, the removal of C3 cladinose caused a remarked decrease (about 10 folds) in antiproliferative activity, which was consistent with the SAR conclusion in our previous study.

**Fig 2:**
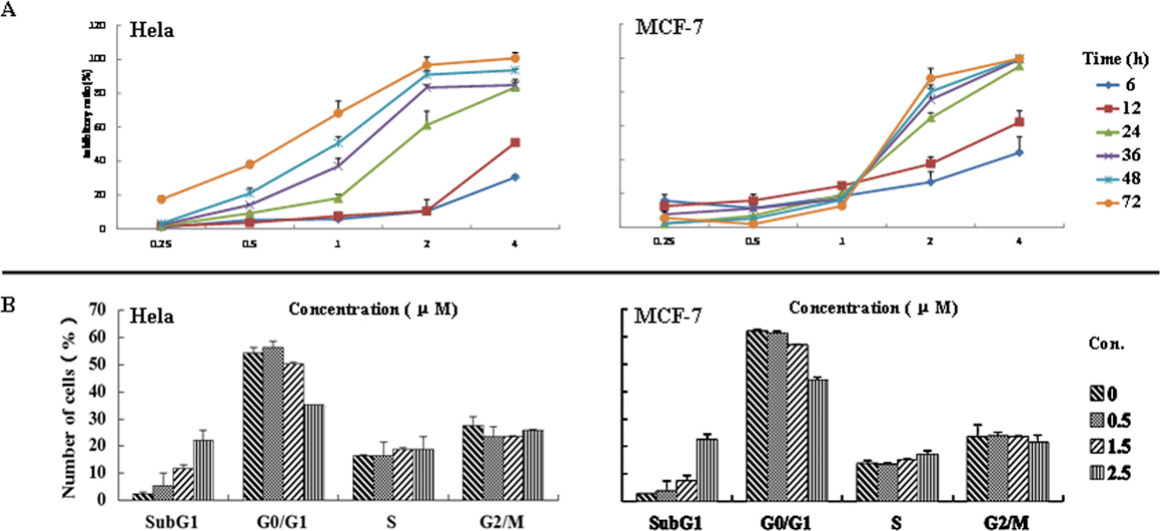
Inhibitory and apoptosis effects of compound 1b on cell proliferation in Hela and MCF-7 cells. (A) Inhibitory effects of compounds **1b**. The cells were treated with various doses of **1b** for 6, 12, 24, 36, 48, 72 h. The inhibitory ratio was measured by MTT assay. (B) Apoptosis effects of compound **1b**. The cells were cultured with 0.5, 1.5, 2.5 μM **1b** for 24 h, stained with PI at 4°C for 30 min, and measured by flow cytometry after collection. The percentage of cells in different phases of the cell cycle was represented by a bar diagram. All of the values were mean±SD of 3 separate experiments.

To investigate the scope of antiproliferative activity of the prepared compounds, *in vitro* antiproliferative activity of the selected most active compound, **1b**, against six other human tumor cell lines, including non-small cell lung carcinoma (A549), gastric carcinoma (BGC-823), hepatocellular carcinoma (HepG-2), laryngeal carcinoma (Hep2), breast carcinoma (MCF-7), cervical carcinoma (HeLa), was determined using MTT assay. As shown in Table 2, compound **1b** showed broad-spectrum antiproliferative activity against the six tumor cell lines with the IC_50_ around 1.5 μM. Further MTT assay for the treatment of Hela and MCF-7 cells with **1b** indicated that the inhibitory effects depended on the concentration of **1b** and the length of time of treatment (Fig 2A).

**Table 2:** In vitro antiproliferative activities of compound 1b against six human cancer cell lines.

Compd.	IC_50_ μM ^a^	
	1b	Cpt
**BGC-823**	1.0±0.2	11.8±0.2
**A549**	3.3±0.2	5.8±0.2
**HepG-2**	1.9±0.4	4.7±0.4
**Hep2**	0.8±0.2	12.3±1.3
**HeLa**	1.2±0.1	17.4±1.4
**MCF-7**	1.1±0.1	2.9±0.1

^a^ IC_50_, expressed as the concentration of drug inhibiting cell growth by 50% after 24 h of drug exposure. All of the values were mean±SD of 3 separate experiments.

#### Flow cytometry for cell cycle analysis

To understand the mechanism driving the antiproliferative activity of the prepared compounds, we analyzed using flow cytometry the cell cycles of Hela and MCF-7 treated for 24h with the selected active compound **1b** at 0.5, 1.0 and 2.0μM concentrations (Fig 2B). It was clear to see that the percentage of SubG_1_ peaks increased from 2.28% (control) to 20.06% for Hela cells, and 3.62% (control) to 22.51% for MCF-7 cells, while the percentage of G_0_/G_1_ peaks decreased accordingly from 55% (control) to 35% for Hela cells, and 61% (control) to 45% for MCF-7 cells. The percentage of apoptotic cells depended on the concentration of **1b**. These results indicated that the antiproliferative activity of **1b** may relate to the induction of cell apoptosis.

Compared to the EA-dimers with a two-atom linker that caused the HT-1080 cells arrested at G_0_/G_1_ phase in our previous study, conversion to the four-atom linker maintained the antiproliferative activity of the EA-dimers while changing the cell death pathway and mechanism.

#### Cell morphology and DNA fragmentation observation

To confirm the antiproliferative activity of **1b** and the fact that it caused the cycle arrest of Hela and MCF-7 cells, effects of **1b** on cellular morphology were examined using immunofluorescence.

**Fig 3:**
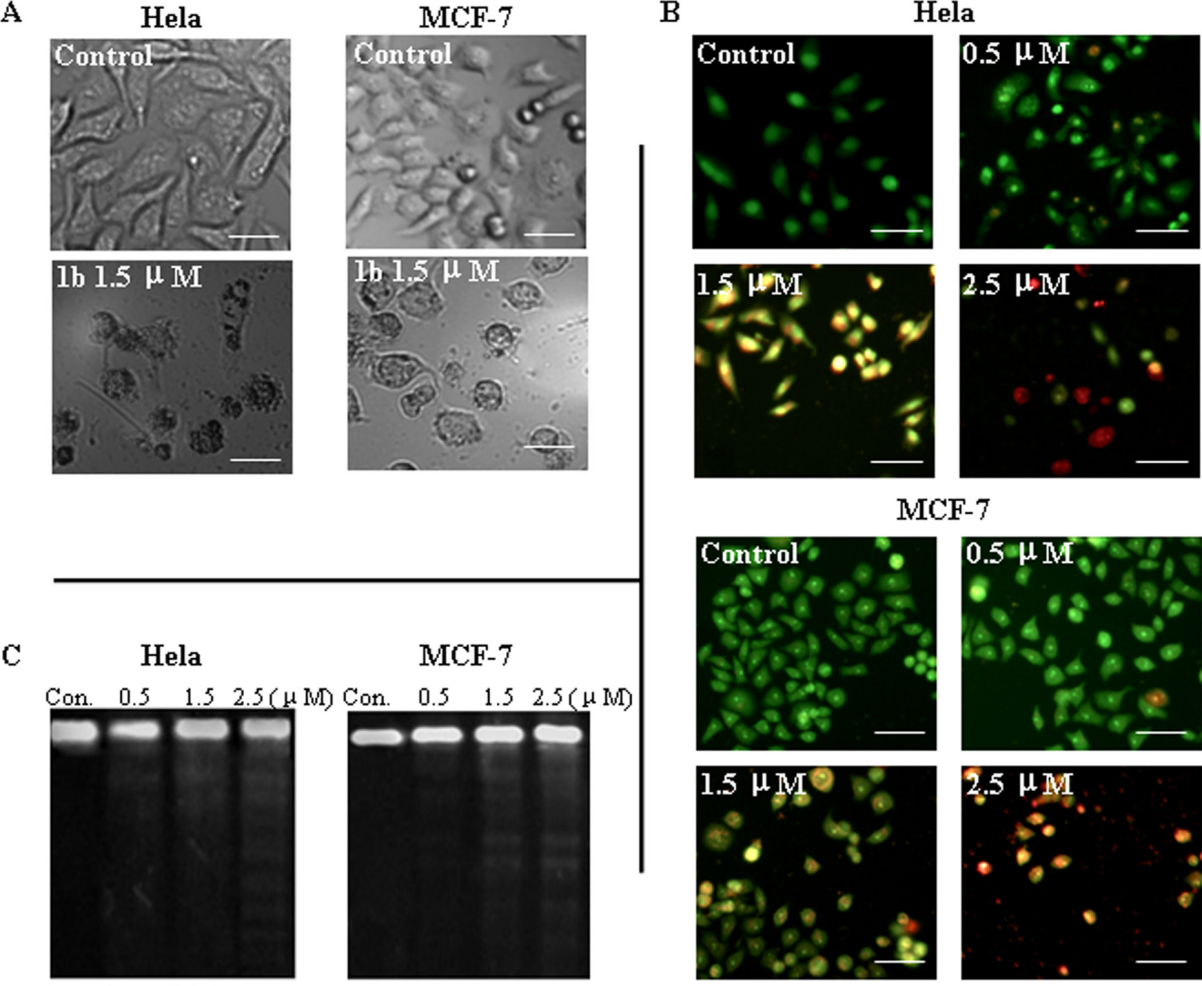
Apoptosis and DNA fragmentation induced by compound 1b on HeLa and MCF-7 cells. (A) Cell morphology observation under a phase contrast microscopy after incubation with medium or 1.5 μM of compound **1b** for 24 h. (B) Cellular morphologic observation under a fluorescence microscopy by AO-EB staining. (C) DNA fragmentation observation. (Scale bar = 10 μm)

As shown in Fig 3, the Hela and MCF-7 cells of the scaffold grew normally in accordance with close-packed rule in the form of polygon. However, upon exposure to **1b** for 24 h, the cells became sparse and changed to round shape, and were accompanied by the formation apoptotic body. With the acridine orange-ethidium bromide (AO/EB) fluorescent staining, control cells appeared to be round, intact and bright green while the exposed cells exhibited irregular morphology and condensed nucleus. In addition, the number of early apoptotic (yellow-green color for 1.5 μM) and late apoptotic (orange color for 2.5 μM) cells rose with the increase of the concentration of **1b**.

DNA fragmentation is an important feature of cell apoptosis. In our test, the increase of apoptotic cells was also reflected in changes in the DNA ladder of **1b** treated Hela and MCF-7 cells. These results plus the cellular morphology alterations confirmed that the antiproliferative mechanism of **1b** relates to the induction of cell apoptosis.

#### Annexin V-FITC apoptosis analysis

**Fig 4:**
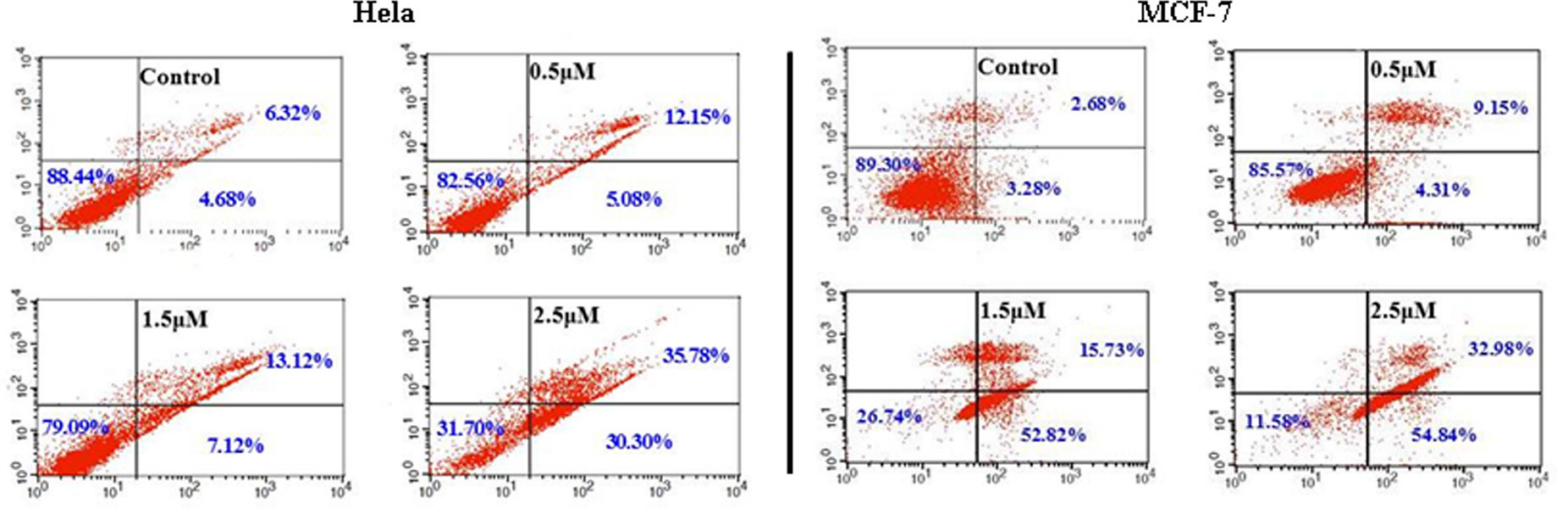
Flowcytometric analysis of AnnexinV binding to the membrane phospholipid phosphatidylserine in Hela and MCF-7 cells. After treatment with 0.5, 1.5, 2.5 μM of compound **1b** for 24h, the cells were stained with AnnexinV-FITC and propidium iodine, and then subjected to the flow cytometric analysis.

As shown in Fig 4, control and **1b** treated (0.5, 1.5 and 2.5 μM) Hela and MCF-7 cells were stained using the Annexin V FITC apoptosis detection kit, respectively. The majority of cells (Hela, 88.44% and MCF-7 89.30%) in the control samples were viable and non-apoptotic. In contrast, when cells were treated with 2.5 μM of **1b** for 24 hours, 31.70% and 11.58% Annexin V-PI- Hela and MCF-7 cells were observed. There was an increase in early apoptotic cell populations from control to 2.5 μM **1b** treated cells (Hela, 4.68% to 30.30% and MCF-7 3.28% to 54.84%). In addition, an increase in the Annexin V+PI+ population was also observed which indicates late apoptotic or dead cells. These observations were consistent with the results showed in Fig 3.

#### Mitochondrial potential alternation

**Fig 5:**
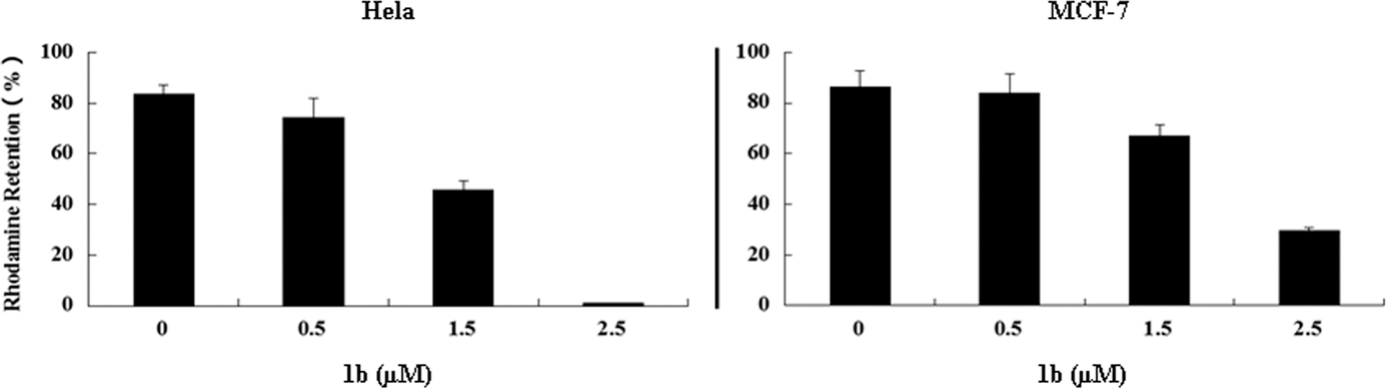
Mitochondrial potential alternation of HeLa and MCF-7 cells by compound 1b. After treatment with 0.5, 1.5, 2.5 μM of **1b** for 24h, the cells were stained with 5g/L Rhodamine 123. Fluorescent density reflected mitochondrial transmembrane potential was determined by flow cytometric analysis. Values were mean±SD of 3 separate experiments.

Mitochondria play a key role in the process of apoptosis. After treatment with **1b** for 24h and stained with Rhodamine 123, the mitochondrial membrane potential (Δψm) changes in HeLa and MCF-7 cells were observed by flow cytometer. As shown in Fig 5, with the increase of the **1b**’s concentration from 0.5 to 2.5 μM, the membrane potential of HeLa cells decreased from 74.17% to 1.00%. Similar trend was obtained in the observation of potential changes in MCF-7 cells and in indicated that **1b** treatment changed the mitochondrial membrane potential in a dose-dependent manner, and the apoptosis of HeLa and MCF-7 cells might be associated with the mitochondrial pathway.

#### Western blotting analysis

**Fig 6:**
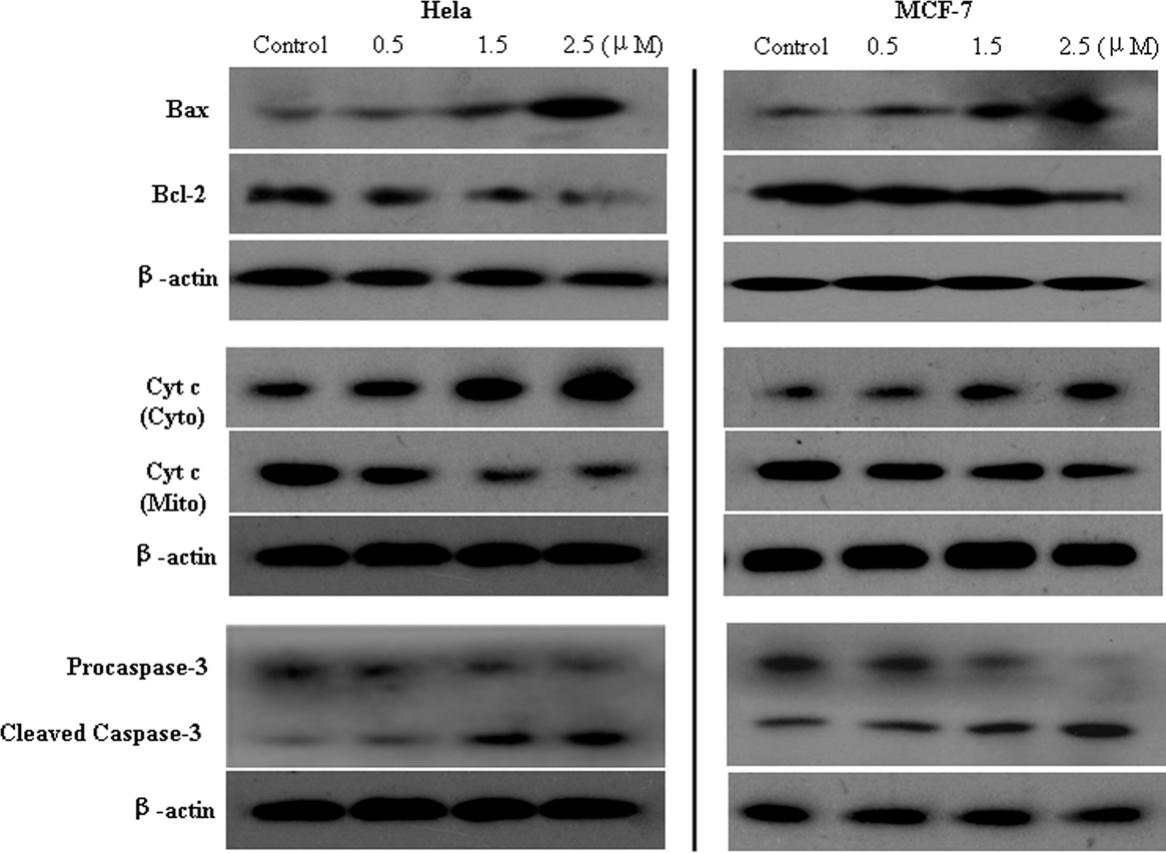
Expression of Bax, Bcl-2, translocation of Cyt c and Capase-3 in HeLa and MCF-7 cells. The cells were treated with different concentration of **1b** (0, 0.5, 1.5 and 2.5 μM) for 24h.

Bax and Bcl-2 proteins are two key apoptosis regulators of Bcl-2 family that govern mitochondrial outer membrane permeabilization (MOMP). To investigate the effects of **1b** on Bax and Bcl-2 expressions and its association with cell apoptosis and proliferation, HeLa and MCF-7 cells were treated with different concentration of **1b** for 24h before western blot analysis (Fig 6). Compared with the control groups, **1b** significantly increased Bax expression and decreased BCL-2 expression both in HeLa and MCF-7 cells, in a dose-dependent manner (P<0.01). Treatment with **1b** led to Δψm depolarization, MOMP enhancing and pro-apoptotic proteins release.

Cytochrome complex (cyt c) and Caspase-3 play important roles in the process of cell apoptosis. In our case, the effects of **1b** on the expression of cyt c and Caspase-3 were examined by treatment of HeLa and MCF-7 cells with different concentration of **1b**. As shown in Fig 6, the expression of cyt c in the cytoplasm increased when the concentration of **1b** increased from 0.5 to 2.5μM. Treatment with **1b** promoted the translocation of cyt c from the mitochondria to cytoplasm. In addition, the amount of 17 kDa cleaved Caspase-3 increased, accompanying with the reducing of zymogen. Although the exact target of compound **1b** is unclear at this point, the above results indicated that the antiproliferative mechanism of **1b** may involve the induction of apoptosis in activating the mitochondrial pathway.

## Conclusions

In conclusion, a series of dimers of de(N-methyl) EM-A derivatives jointed by a four-atom linker, -CH_2_CH = CHCH_2_-, were prepared and their antiproliferative activity against SGC-7901 gastric carcinoma, HT-1080 fibrosarcoma carcinoma and KB oral squamous carcinoma was evaluated. SAR study indicated that 1) transformation of the two-atom linker into four-atom linker lead to an increase in potency for compounds with 14-membered lactone rings. 2) Removal of C3 cladinose resulted in a marked decrease in activity. 3) Conversion of the C9 carbonyl to O-alkyloxime groups had limited influence on activity. 4) Among the EM-A dimers synthesized, compound **1b** carrying C6 methoxyl groups showed potent activity against the three cell lines and six other cell lines in further MTT assay.

From flow cytometry analysis on HeLa and MCF-7 cells, we concluded that the four-atom EM-A dimers induced the SubG_1_ phase cell cycle arrest and cell apoptosis, in time- and dose-dependent manners. Preliminary pharmacological experiments including morphologic observation, DNA agarose gel electrophoresis, mitochondrial potential alternation and western blot analysis revealed that the antiproliferative mechanism may involve the induction of apoptosis in activating the mitochondrial pathway, and regulation of apoptotic proteins. More SAR and detailed mechanism studies on the antiproliferative activity are under way and will be reported in due course.

## Materials and Methods

### Chemistry

All chemicals and solvents were of American Chemical Society grade or HPLC purity. Sigma-Aldrich (Beijing, China) and other commercially available sources are the sources for the starting materials utilized in the presented synthesis and the reagents were used without purification. Organic solvents were dried by standard methods when necessary. Thin-layer chromatography was performed on GF254 silica gel plates to monitor the reaction and the plates were examined under UV light or detected with a solution of phosphomolybdic acid in ethanol (5%). The purification of the products was performed using column chromatography (60 Å, 200–300 mesh, Qingdao Ocean Chemicals) or silica gel plates (0.25mm layer, Qingdao Ocean Chemicals) with the designated solvents. Mass spectra were obtained on a Waters Quattro Micro API or Agilent 1100 series MSD TRAP using ESI. Elemental analyses were performed on a vario MACRO cube CHNS element analyzer (ELEMENTAR Analysensysteme, Hanau, Germany). ^1^H and ^13^C NMR spectra were taken in CDCl_3_ solution on Bruker ARX-300 spectrometers with TMS as the internal reference. Chemical shifts were reported in ppm downfield from tetramethylsilane.

The target compounds were synthesized by alkylation of the de(N-methyl) EM-A derivatives 3a–3m (S1 Fig). Compounds **3a**–**3k** were obtained, starting from EM-A, clarithromycin and other EM-A derivatives modified at the C9 position followed by N-demethylation with iodine and sodium acetate in methanol [21]. The cladinose moieties were selectively removed upon treatment with dilute hydrochloric acid in aqueous solution before N-demethylation to give compounds **3i** and **3j** [22]. Compounds **3l** and **3m** were synthesized according to the methods reported methods [23], followed by N-demethylation. See **Supporting Information** for detailed chemical syntheses.

### Bioassay

#### Reagent and Cell culture

AnnexinV-FITC/PI Assay Kit and Cell Mitochondria Isolation Kit were purchased from Beyotime Institute of Biotechnology, Jiangsu, China; DNA Ladder Detection Kit from Dingguochangsheng Biotechnology, Beijing, China; Mouse β-actin, Rabbit Bax, Rabbit Bcl-2, Rabbit caspase-3, Rabbit Cytochrome-C were purchased from Proteintech, Wuhan, China.

All cell lines were purchased from American Type Culture Collection (ATCC, Manassas, VA, USA). Non-small cell lung carcinoma cells (A549), hepatocellular carcinoma cells (HepG-2) and breast carcinoma cells (MCF-7) were cultured in DMEM medium (Sigma, St. Louis, MO, USA), while gastric carcinoma cells (BGC-823), laryngeal carcinoma cells (Hep2), cervical carcinoma cells (HeLa) were cultured in RPMI-1640 medium (Sigma, St. Louis, MO, USA), which were supplemented with 10% fetal bovine serum (FBS; TBD, Tianjin, China) at 37°C in humidified atmosphere with 5% CO_2_.

#### MTT assay

The antiproliferative activity of the synthetic compounds was measured by 3-(4, 5- dimethylthiazol-2-yl)-2, 5-diphenyltetrazolium bromide (MTT) assay. Cells were seeded in 96-well plates (Corning, NY, USA) at a density of 8×10^3^ per well. After 24 h of incubation, cells were treated with different concentrations (0.1, 0.3, 1, 3 and 10 μg/ml) of the EM-A dimers for the indicated time periods. Afterwards, MTT (Sigma, St. Louis, MO) solution [5.0 mg/ml in phosphate-buffered saline (PBS)] was added (20 μl/well) and incubated for another 4 h at 37°C. The purple formazan crystals were then dissolved in 100 μl dimethyl sulfoxide (DMSO). After 5 min, the plates were read on a plate microreader (TECANSPECTRA, Wetzlar, Germanay) at 490 nm. The IC_50_ values were obtained using the software of Dose–Effect Analysis with Microcomputers and were defined as concentration of drug causing 50% inhibition in absorbance compared with control cells. Assays were performed in triplicate from three independent experiments.

#### Cell cycle analysis

Cells were dispensed in 25 ml culture bottle at a density of 5 ×10^5^ per bottle. After 24 h incubation, they were treated with compound **1b** at given concentrations for 24h. The cells were harvested by 0.05% trypsin (Sigma, St. Louis, MO), then collected by centrifugation, washed with PBS, and fixed with 10 ml ice-cold 70% ethanol at 4°C overnight. After washed with PBS, the cells were suspended in 1ml propidium iodide (PI; 50 mg/L; Sigma, St. Louis, MO) solution supplemented with 1 g/L RNase A (Sigma, St. Louis, MO). Finally, the samples were analyzed by FACScan flow cytometer (Becton Dickinson, Franklin, NJ, USA).

#### Cell morphology observation and DNA fragmentation

Cells were seeded in 6-well plates at a density of 1.2×10^5^ (MCF-7) or 1.5×10^5^ (HeLa) per well. After 24 h of incubation, cells were treated with 0 or 0.15 μM **1b** for another 24h and then observed under a phase contrast microscope.

Cells were seeded in 6-well plates at a density of 0.8×10^5^ (MCF-7) or 1.0×10^5^ (HeLa) per well. After 24 h of incubation, cells were treated with 0, 0.5, 1.5 or 2.5 μM **1b** for another 24h. Subsequently, cells were washed with PBS twice, stained with AO-EB working solution, and observed under a fluorescent microscope (Olympus, Tokyo, Japan).

Cells were seeded in 6-well plates at a density of 1.2×10^5^ (MCF-7) or 1.5×10^5^ (HeLa) per well. After 24 h of incubation, cells were treated with 0, 0.5, 1.5 or 2.5 μM **1b** for another 24h. DNA ladder detection kit was used to extract the DNA, and 30 μL of DNA sample was loaded onto a 0.8% agarose gel which was run at 5 V/cm for 1.5 h. The stained DNA was observed by transillumination with UV light and photographed.

#### Annexin V-FITC apoptosis analysis

Cell apoptosis was determined using AnnexinV-FITC/PI Assay Kit according to the manufacturers’ protocols. Briefly, cells were seeded in 6-well plates at a density of 1.2×10^5^ (MCF-7) or 1.5×10^5^ (HeLa) per well and incubated for 24 h. Then, cells were treated with 0, 0.5, 1.5 or 2.5 μM **1b** for 24h. After washed with PBS twice, cells were re-suspended in 100 μL binding buffer containing 5 μL AnnexinV-FITC and 5 μL PI. Following 10 min of incubation, cells were detected by FACScan flow cytometer.

#### Mitochondrial potential assay

Cells were seeded in 6-well plates at a density of 0.8×10^5^ (MCF-7) or 1.0×10^5^ (HeLa) per well. After 24 h of incubation, cells were treated with 0, 0.5, 1.5 or 2.5 μM **1b** for another 24h. Subsequently, cells were washed with PBS twice, incubated with 1 μg/mL Rhodamine 123 in 1 mL PBS at 37°C for 30 min, and analyzed by FACScan flow cytometer.

#### Western blot analysis

Cells were seeded in 75 mL culture flask at a density of 8 ×10^5^ (MCF-7) or 1.2×10^6^ (HeLa) and incubated for 24 h. Thereafter, cells were treated with 0, 0.5, 1.5 or 2.5 μM **1b** for 24h and harvested by trypsin into the EP tubes.

For total protein extraction, the cell pellets were lysed in RIPA buffer (Beyotime) supplemented with PMSF on ice for 30 min. After centrifuging at 12000 g for 10 min at 4°C, the cell suspension was collected as the whole cell protein. Cytoplasmic proteins and mitochondrial proteins were extracted from cells using Cell Mitochondria Isolation Kit according to the instructions of manufacturer. The protein concentrations were quantified with BCA Protein Assay Kit (Beyotime) and a plate reader according to the manufacturer’s protocols.

For western blot analysis, equal protein lysates were separated by electrophoresis on 12% SDS-PAGE gels, and transferred onto PVDF membranes. After blocking with 5% non-fat milk in PBS for 2.5 h at room temperature, the membranes were incubated with primary antibody overnight at 4°C: Bax (1:2000), Bcl-2 (1:1000), Caspase-3 (1:500), Cytochrome c (1:500), and β-actin (1:3000). Then, the blots were washed three times for 10 min each in Tris-NaCl and incubated with secondary HRP-conjugated goat anti-mouse or anti-rabbit IgGs (1:6000 or 1:5000) for 2.5 h at 37°C. The interest proteins were visualized using ECL, and β-actin served as the internal control.

#### Statistical analysis

Data were expressed as mean ± SD (standard deviation) from three independent experiments. Student’s *t* tests were used to compare the means of two groups. p < 0.05 was considered as statistical significance.

## Supporting Information

S1 Fig Synthetic route for the dimers of de(N-methyl) EM-A derivatives. Reagent and conditions: (a) 1,4-bromo-2-butene (0.5 equiv.), DIPEA, CH_2_Cl_2_, r.t.; (b) **3b**, **3e** or **3k** (1.0 equiv.), 1,4-bromo-2-butene (1.0 equiv.), DIPEA, CH_2_Cl_2_, r.t.
(DOC)
