# Supplementary material for: Development of Novel Erythromycin Derivatives with Inhibitory Activity against Proliferation of Tumor Cells
Source: PLoS One. 2016 Jul 22;11(7):e0159503. doi: 10.1371/journal.pone.0159503 (PMC4957748; doi:10.1371/journal.pone.0159503)
Supplement: S1 Fig — Reagent and conditions: (a) 1,4-bromo-2-butene (0.5 equiv.), DIPEA, CH2Cl2, r.t.; (b) 3b, 3e or 3k (1.0 equiv.), 1,4-bromo-2-butene (1.0 equiv.), DIPEA, CH2Cl2, r.t. (DOC) [file pone.0159503.s001.doc]

**Development of novel erythromycin derivatives with inhibitory activity against proliferation of tumor cells**

Lan Wu^1^*, Kai Bao^2,3^, Rui Song^4^, Defa Wang^3^, Lei Zhang^1^, Weiyun Wang^4^, Weige Zhang^3^, Wen Bin^4^*

1 Department of Geratology, The First Afﬁliated Hospital of Chinese Medical University, Shenyang, China

2 State Key Laboratory of Bioactive Substance and Function of Natural Medicines, Institute of Materia Medica, Chinese Academy of Medical Sciences and Peking Union Medical College, Beijing, China

3 Key Laboratory of Structure-Based Drug Design and Discovery Ministry of Education, Shenyang Pharmaceutical University, Shenyang, China

4 School of Life Sciences and Biopharmaceutics, Shenyang Pharmaceutical University, Shenyang, China

Supporting Information – Table of Contents

**S1 Fig**. Synthetic route for the dimers of de(N-methyl) EM-A derivatives**................2**

**Materials and Methods ............................................................................................ 3**

**Experimental Procedures ........................................................................................ 4**

**S1 Fig**. **Synthetic route for the dimers of de(N-methyl) EM-A derivatives.** Reagent and conditions: (a) 1,4-bromo-2-butene (0.5 equiv.), DIPEA, CH_2_Cl_2_, r.t.; (b) **3b**, **3e** or **3k** (1.0 equiv.), 1,4-bromo-2-butene (1.0 equiv.), DIPEA, CH_2_Cl_2_, r.t.

**Materials and Methods.**

**Chemistry**

All chemicals and solvents were of American Chemical Society grade or HPLC purity. Sigma-Aldrich (Beijing, China) and other commercially available sources are the sources for the starting materials utilized in the presented synthesis and the reagents were used without purification. Organic solvents were dried by standard methods when necessary. Thin-layer chromatography was performed on GF254 silica gel plates to monitor the reaction and the plates were examined under UV light or detected with a solution of phosphomolybdic acid in ethanol (5%). The purification of the products was performed using column chromatography (60 Å, 200-300 mesh, Qingdao Ocean Chemicals) or silica gel plates (0.25mm layer, Qingdao Ocean Chemicals) with the designated solvents. Mass spectra were obtained on a Waters Quattro Micro API or Agilent 1100 series MSD TRAP using ESI. Elemental analyses were performed on a vario MACRO cube CHNS element analyzer (ELEMENTAR Analysensysteme, Hanau, Germany). ^1^H and ^13^C NMR spectra were taken in CDCl_3_ solution on Bruker ARX-300 spectrometers with TMS as the internal reference. Chemical shifts were reported in ppm downfield from tetramethylsilane.

**1a**

1,4-bromo-2-butene (15.0 mg, 0.07 mmol) was added to the solution of compound **3a** (0.1 g, 0.14 mmol) and *N,N*-diisopropylethylamine (0.07 mL, 0.42 mmol) in dichloromethane (5.0 mL) at room temperature. The reaction mixture was stirred at room temperature for 120 h. The mixture was poured into water and extracted with dichloromethane. The extract was washed with brine, dried over Na_2_SO_4_, filtered, and concentrated *in vacuo*. The crude product was purified by preparative thin layer chromatography with chloroform-methanol-ammonium hydroxide solution (20:1:0.1) to yield **1a** (76.2 mg, 73%) as a white solid. ^13^C NMR (75 MHz, CDCl_3_): δ 221.7 (C-9), 175.7 (C-1), 103.1 (C-1'), 96.3 (C-1''), 83.5 (C-5), 79.9 (C-3), 77.9 (C-4''), 76.8 (C-13), 75.0 (C-6), 74.6 (C-12), 72.6 (C-3''), 70.8 (C-2'), 68.9 (C-11), 68.8 (C-5'), 65.5 (C-5''), 64.9 (C-3'), 55.6 (NCH_2_), 49.5 (3''-OMe), 45.2 (C-2), 44.9 (C-8), 39.3 (C-4), 38.5 (C-7), 37.7 (C-10), 36.5 (NMe), 34.9 (C-2''), 29.7 (C-4'), 27.0 (C-6 Me), 21.5 (C-5' Me), 21.4 (C-3'' Me), 21.3 (C-14), 18.6 (C-5'' Me), 18.2 (C-8 Me), 16.1 (C-12 Me), 16.0 (C-2 Me), 12.0 (C-10 Me), 10.6 (C-15), 9.2 (C-4 Me), 130.3 (CH=CH); ^1^H NMR (300 MHz, CDCl_3_): δ 5.53 (CH=CH), 4.97 (H-13), 4.82 (H-1''), 4.34 (H-1'), 3.25 (3''-OMe), 2.16 (NMe); MS (ESI, *m/z*): 1491.8 [M+H^+^], 1526.0 [M+Cl^−^]. Anal. Calcd. for C_76_H_134_N_2_O_26_: C, 61.19; H, 9.05; N, 1.88. Found: C, 61.17; H, 9.00; N, 1.92.

**1b**

White solid, yield 76%; ^13^C NMR (75 MHz, CDCl_3_): δ 221.1 (C-9), 175.8 (C-1), 102.7 (C-1'), 96.0 (C-1''), 80.8 (C-5), 78.4 (C-6), 78.3 (C-3), 77.9 (C-4''), 76.5 (C-13), 74.2 (C-12), 72.6 (C-3''), 70.8 (C-2'), 69.0 (C-11), 68.7 (C-5'), 65.7 (C-5''), 65.0 (C-3'), 55.6 (NCH_2_), 50.6 (6-OMe), 49.5 (3''-OMe), 45.2 (C-2), 45.0 (C-8), 39.3 (C-7), 39.1 (C-4), 37.2 (C-10), 36.5 (NMe), 34.9 (C-2''), 29.8 (C-4'), 21.4 (C-5' Me/C-3'' Me), 21.0 (C-14), 19.7 (C-6 Me), 18.7 (C-5'' Me), 18.0 (C-8 Me), 16.0 (C-12 Me), 15.9 (C-2 Me), 12.3 (C-10 Me), 10.6 (C-15), 9.0 (C-4 Me), 130.9 (CH=CH); ^1^H NMR (300 MHz, CDCl_3_): δ 5.59 (CH=CH), 5.04 (H-13), 4.92 (H-1''), 4.42 (H-1'), 3.31 (3''-OMe), 3.03 (6-OMe), 2.20 (NMe); MS (ESI, *m/z*): 1519.7 [M+H^+^], 1554.2 [M+Cl^−^]. Anal. Calcd. for C_78_H_138_N_2_O_26_: C, 61.64; H, 9.15; N, 1.84. Found: C, 61.55; H, 9.18; N, 1.84.

**1c**

White solid, yield 69%; ^13^C NMR (75 MHz, CDCl_3_): δ 174.7 (C-9), 173.6 (C-1), 102.5 (C-1'), 96.8 (C-1''), 83.6 (C-5), 79.9 (C-3), 77.4 (C-4''), 77.0 (C-13), 75.2 (C-6), 74.8 (C-12), 74.2 (C-3''), 70.5 (C-2'/C-11), 68.0 (C-5'), 62.8 (C-3'/C-5''), 55.9 (NCH_2_), 50.9 (3''-OMe), 44.6 (C-2), 38.7 (C-4), 37.9 (C-7), 36.6 (NMe), 35.0 (C-2''), 31.9 (C-10), 29.7 (C-4'), 29.4 (C-8), 26.4 (C-6 Me), 22.7 (C-3'' Me), 21.5 (C-5' Me), 21.1 (C-14), 19.1 (C-8 Me), 18.7 (C-5'' Me), 16.3 (C-2 Me/C-12 Me), 14.5 (C-10 Me), 10.6 (C-15), 9.1 (C-4 Me), 130.9 (CH=CH), 133.6, 128.8 (OAllyl); ^1^H NMR (300 MHz, CDCl_3_): δ 5.90, 5.31, 5.23 (OAllyl), 5.59 (CH=CH), 5.18 (H-13), 4.90 (H-1''), 4.49 (H-1'), 3.35 (3''-OMe), 2.17 (NMe); MS (ESI, *m/z*): 1602.7 [M+H^+^]. Anal. Calcd. for C_83_H_141_N_3_O_26_: C, 61.48; H, 9.06; N, 3.50. Found: C, 61.47; H, 9.18; N, 3.62.

**1d**

White solid, yield 74%; ^13^C NMR (75 MHz, CDCl_3_): δ175.2 (C-1), 173.3 (C-9), 103.0 (C-1'), 96.3 (C-1''), 83.3 (C-5), 80.0 (C-3), 78.0 (C-4''), 76.9 (C-13), 75.2 (C-6), 74.3 (C-12), 72.7 (C-3''), 70.8 (C-2'), 70.5 (C-11), 68.8 (C-5'), 65.6/65.5 (C-3'), 64.9 (C-5''), 55.6 (NCH_2_), 49.5 (3''-OMe), 44.7 (C-2), 39.0 (C-4), 37.7 (C-7), 36.5 (NMe), 35.1 (C-2''), 33.1 (C-10), 30.6 (C-4'), 27.0 (C-8/C-6 Me), 21.5 (C-3'' Me), 21.4 (C-5' Me), 21.1 (C-14), 18.7 (C-8 Me), 18.6 (C-5'' Me), 16.3 (C-2 Me), 16.1 (C-12 Me), 14.5/13.7 (C-10 Me), 10.7 (C-15), 9.2 (C-4 Me), 98.9, 57.2 (OCH_2_OCH_3_), 130.9 (CH=CH); ^1^H NMR (300 MHz, CDCl_3_): δ 5.59 (CH=CH), 5.05 (H-13), 4.91 (H-1''), 4.41 (H-1'), 3.32 (3''-OMe), 2.22 (NMe), 5.13, 3.44 (OCH_2_OCH_3_); MS (ESI, *m/z*): 1610.7 [M+H^+^], 1633.8 [M+Na^+^], 1608.0 [M- H^+^]. Anal. Calcd. for C_80_H_144_N_4_O_28_: C, 59.68; H, 9.02; N, 3.48. Found: C, 59.65; H, 9.04; N, 3.47.

**1e**

White solid, yield 71%; ^13^C NMR (75 MHz, CDCl_3_): δ 175.0 (C-1), 172.7 (C-9), 102.9 (C-1'), 96.2 (C-1''), 83.1 (C-5), 80.3 (C-3), 78.0 (C-4''), 76.8 (C-13), 74.7 (C-6), 74.3 (C-12), 72.8 (C-3''), 71.9 (C-2'), 70.4 (C-11), 68.5 (C-5'), 65.6 (C-3'), 65.5 (C-5''), 56.0 (NCH_2_), 49.5 (3''-OMe), 44.7 (C-2), 38.8 (C-4), 37.5 (C-7), 36.3 (NMe), 35.1 (C-2''), 33.0 (C-10), 30.6 (C-4'), 26.9 (C-8), 26.8 (C-6 Me), 21.5 (C-3'' Me), 21.2 (C-5' Me), 21.1 (C-14), 18.8 (C-8 Me), 18.6 (C-5'' Me), 16.3 (C-2 Me), 16.2 (C-12 Me), 14.8 (C-10 Me), 10.6 (C-15), 9.4 (C-4 Me), 97.5, 71.9, 68.3, 59.1 (OCH_2_OCH_2_CH_2_OCH_3_), 130.9/ 128.9 (CH=CH); ^1^H NMR (300 MHz, CDCl_3_): δ 5.81 (CH=CH), 5.13 (H-13), 4.87 (H-1''), 4.31 (H-1'), 3.31 (3''-OMe), 2.38 (NMe), 5.19, 3.74, 3.58, 3.42 (OCH_2_OCH_2_CH_2_OCH_3_); MS (ESI, *m/z*): 1699.0 [M+H^+^], 1721.5 [M+Na^+^], 1732.8 [M+Cl^−^]. Anal. Calcd. for C_84_H_152_N_4_O_30_: C, 59.41; H, 9.02; N, 3.30. Found: C, 59.44; H, 8.97; N, 3.26.

**1f**

White solid, yield 63%; ^13^C NMR (75 MHz, CDCl_3_): δ175.1 (C-1), 171.3 (C-9), 102.8 (C-1'), 96.2 (C-1''), 83.1 (C-5), 79.9 (C-3), 77.9 (C-4''), 77.0 (C-13), 74.2 (C-6), 74.1 (C-12), 72.7 (C-3''), 70.8 (C-2'), 70.5 (C-11), 68.7 (C-5'), 65.5 (C-3'), 64.8 (C-5''), 55.6 (NCH_2_), 49.4 (3''-OMe), 44.6 (C-2), 38.9 (C-4), 37.7 (C-7), 36.5 (NMe), 35.0 (C-2''), 32.8 (C-10), 29.9 (C-4'), 27.0 (C-8), 26.3 (C-6 Me), 21.4 (C-3'' Me), 21.3 (C-5' Me), 21.0 (C-14), 18.6 (C-8 Me), 18.5 (C-5'' Me), 16.2 (C-2 Me/C-12 Me), 14.4 (C-10 Me), 10.6 (C-15), 9.1 (C-4 Me), 130.9 (CH=CH), 75.3, 29.9, 28.5, 28.1, 14.0 (OPentyl); ^1^H NMR (300 MHz, CDCl_3_): δ 5.59 (CH=CH), 5.13 (H-13), 4.92 (H-1''), 4.43 (H-1'), 3.32 (3''-OMe), 2.22 (NMe); MS (ESI, *m/z*): 1662.7 [M+H^+^], 842.7 [M/2+Na^+^]. Anal. Calcd. for C_86_H_156_N_4_O_26_: C, 62.14; H, 9.46; N, 3.37. Found: C, 62.14; H, 9.41; N, 3.35.

**1g**

White solid, yield 71%; ^13^C NMR (75 MHz, CDCl_3_): δ 175.8 (C-1), 172.9 (C-9), 103.5 (C-1'), 96.9 (C-1''), 83.9 (C-5), 80.5 (C-3), 78.6 (C-4''), 77.0 (C-13), 74.9 (C-6), 73.4 (C-12), 71.5 (C-2'/C-3''), 71.2 (C-11), 69.3 (C-5'), 66.1 (C-3'), 65.5 (C-5''), 56.8 (NCH_2_), 50.1 (3''-OMe), 45.3 (C-2), 39.6 (C-4), 38.3 (C-7), 37.1 (NMe), 35.7 (C-2''), 33.7 (C-10), 30.7/30.0 (C-4'), 27.8 (C-8), 27.3 (C-6 Me), 22.7 (C-3'' Me), 22.4/22.1 (C-5' Me), 22.0/21.8 (C-14), 19.3 (C-8 Me), 19.2 (C-5'' Me), 16.9 (C-2 Me), 15.3 (C-12 Me), 14.8 (C-10 Me), 11.4 (C-15), 9.8 (C-4 Me), 153.9, 138.3, 133.6, 105.8, 76.0, 61.6, 56.3 [O(3,4,5-trimethoxy benzyl)], 131.6 (CH=CH); ^1^H NMR (300 MHz, CDCl_3_): δ 5.59 (CH=CH), 5.10 (H-13), 4.87 (H-1''), 4.40 (H-1'), 3.31 (3''-OMe), 2.24 (NMe), 6.55, 4.98, 3.87, 3.86 [O(3,4,5-trimethoxy benzyl)]; MS (ESI, *m/z*): 1881.6 [M+H^+^], 1915.9 [M+Cl^−^]. Anal. Calcd. for C_96_H_160_N_4_O_32_: C, 61.26; H, 8.57; N, 2.98. Found: C, 61.22; H, 8.54; N, 2.96.

**1h**

White solid, yield 85%; ^13^C NMR (75 MHz, CDCl_3_): δ 175.6 (C-1), 170.4 (C-9), 102.7 (C-1'), 96.0 (C-1''), 80.5 (C-5), 78.7 (C-3), 78.4 (C-4''), 77.9 (C-13), 76.5 (C-6), 74.0 (C-12), 72.7 (C-3''), 71.0 (C-2'), 70.1 (C-11), 68.6 (C-5'), 65.6 (C-3'), 64.8 (C-5''), 55.7 (NCH_2_), 51.1 (6-OMe), 49.4 (3''-OMe), 45.1 (C-2), 39.0 (C-4), 37.4 (NMe), 36.5 (C-7), 34.9 (C-2''), 32.8 (C-10), 30.9 (C-4'), 25.3 (C-8), 21.4 (C-5' Me), 21.3 (C-3'' Me), 21.1 (C-14), 20.0 (C-6 Me), 18.6 (C-5'' Me), 18.5 (C-8 Me), 16.0 (C-2/C-12 Me), 14.9 (C-10 Me), 10.6 (C-15), 9.1 (C-4 Me), 130.9 (CH=CH); ^1^H NMR (300 MHz, CDCl_3_): δ 5.61 (CH=CH), 5.11 (H-13), 4.94 (H-1''), 4.42 (H-1'), 3.33 (3''-OMe), 3.10 (6-OMe), 2.24 (NMe); MS (ESI, *m/z*): 1550.4 [M+H^+^], 1573.3 [M+Na^+^]. Anal. Calcd. for C_78_H_140_N_4_O_26_: C, 60.44; H, 9.10; N, 3.61. Found: C, 60.47; H, 9.01; N, 3.55.

**1i**

White solid, yield 23%; ^13^C NMR (75 MHz, CDCl_3_): δ 221.0/220.7 (C-9), 175.8/175.0 (C-1), 106.6/102.7 (C-1'), 96.0 (C-1''), 88.5/80.7 (C-5), 78.4 (C-3), 78.0 (C-6), 77.2 (C-4''), 76.6 (C-13), 74.2 (C-12), 72.8 (C-3''), 70.9/70.6 (C-2'), 69.8 (C-11), 68.6/68.1 (C-5'), 65.8 (C-5''), 64.9 (C-3'), 55.7 (NCH_2_), 50.6 (6-OMe), 49.5 (3''-OMe), 45.2/44.5 (C-2), 45.1/44.5 (C-8), 39.4/38.7 (C-7), 39.2/36.4 (C-4), 37.5/37.2 (C-10), 36.5 (NMe), 35.9 (C-2''), 30.3/29.4 (C-4'), 21.5 (C-3'' Me), 21.2 (C-5' Me), 21.0 (C-14), 19.8/18.8 (C-6 Me), 18.7 (C-5'' Me), 18.0/17.7 (C-8 Me), 16.2 (C-12 Me), 16.0/15.2 (C-2 Me), 12.6/12.3 (C-10 Me), 10.4/10.2 (C-15), 9.2/8.4 (C-4 Me), 130.7/128.8 (CH=CH); ^1^H NMR (300 MHz, CDCl_3_): δ 5.68 (CH=CH), 5.13 (H-13), 5.00 (H-1''), 4.30 (H-1'), 3.40 (3''-OMe), 3.05 (6-OMe), 2.30 (NMe); MS (ESI, *m/z*): 1362.6 [M+H^+^], 682.1 [M/2+H^+^], 1360.5 [M-H^+^]. Anal. Calcd. for C_70_H_124_N_2_O_23_: C, 61.74; H, 9.18; N, 2.06. Found: C, 61.77; H, 9.15; N, 2.03.

**1j**

White solid, yield 26%; ^13^C NMR (75 MHz, CDCl_3_): δ 175.6/175.0 (C-1), 170.4 (C-9), 106.6/102.7 (C-1'), 96.0 (C-1''), 87.9/80.6 (C-5), 78.7 (C-3), 78.4/77.2 (C-13), 78.3 (C-4''), 76.8 (C-6), 74.1 (C-12), 72.7 (C-3''), 71.0 (C-2'), 70.6/70.2 (C-11), 68.5 (C-5'), 65.6 (C-5''), 65.3/64.7 (C-3'), 55.7 (NCH_2_), 51.2 (6-OMe), 49.5 (3''-OMe), 44.5 (C-2), 39.0/36.1 (C-4), 37.6 (NMe), 37.4 (C-7), 34.9 (C-2''), 32.9 (C-10), 29.7 (C-4'), 25.3/25.2 (C-8), 21.6/21.4 (C-5' Me), 21.5 (C-3'' Me), 21.2 (C-14), 20.0/18.7 (C-6 Me), 18.7 (C-5'' Me), 18.6/18.3 (C-8 Me), 16.3 (C-12 Me), 16.0/15.2 (C-2 Me), 15.1/15.0 (C-10 Me), 10.6/10.5 (C-15), 9.2/8.2 (C-4 Me), 130.6/128.8 (CH=CH); ^1^H NMR (300 MHz, CDCl_3_): δ 5.60 (CH=CH), 5.09 (H-13), 4.94 (H-1''), 4.44/4.37 (H-1'), 3.33 (3''-OMe), 3.10 (6-OMe), 2.27/2.21 (NMe); MS (ESI, *m/z*): 1391.9 [M+H^+^], 1413.9 [M+Na^+^]. Anal. Calcd. for C_70_H_126_N_4_O_23_: C, 60.41; H, 9.13; N, 4.03. Found: C, 60.37; H, 9.11; N, 4.00.

**1k**

1,4-bromo-2-butene (32.1 mg, 0.15 mmol) was added to a solution of compound **5a** (0.11 g, 0.15 mmol), compound **5b** (0.11 g, 0.15 mmol) and *N,N*-diisopropylethylamine (0.07 mL, 0.42 mmol) in anhydrous dichloromethane (5.0 mL) at room temperature. The reaction mixture was stirred at room temperature for 120 h. The mixture was poured into water and extracted with dichloromethane. The extract was washed with brine, dried over Na_2_SO_4_, filtered, and concentrated *in vacuo*. The crude product was purified by preparative thin layer chromatography with chloroform-methanol-ammonium hydroxide solution (20:1:0.1) to yield **1k** (54.2 mg, 24 %) as a white solid. ^13^C NMR (75 MHz, CDCl_3_): δ 221.0/220.9 (C-9), 175.8/175.7 (C-1), 102.8/102.3 (C-1'), 96.1 (C-1''), 83.4/80.9 (C-5), 80.0 (C-3), 78.4/75.0 (C-6), 78.0 (C-4''), 76.8 (C-13), 74.6/74.3 (C-12), 72.7 (C-3''), 70.9 (C-2'), 69.1 (C-11), 68.9/68.7 (C-5'), 65.7 (C-5''), 65.6 (C-3'), 56.8 (NCH_2_), 50.7 (6-OMe), 49.5/49.4 (3''-OMe), 45.2 (C-2), 45.1/44.9 (C-8), 39.4 (C-4), 39.2/39.0 (C-7), 37.2 (C-10), 36.4 (NMe), 34.9 (C-2''), 29.7/29.4 (C-4'), 27.0/19.7 (C-6 Me), 21.5 (C-3'' Me), 21.4 (C-5' Me), 21.2/21.0 (C-14), 18.7/18.6 (C-5'' Me), 18.3/18.0 (C-8 Me), 16.2 (C-12 Me), 16.0 (C-2 Me), 12.3/12.0 (C-10 Me), 10.7/10.6 (C-15), 9.1 (C-4 Me), 130.9/128.8 (CH=CH); ^1^H NMR (300 MHz, CDCl_3_): δ 5.61 (CH=CH), 5.07/5.04 (H-13), 4.92/4.88 (H-1''), 4.43 (H-1'), 3.32/3.31 (3''-OMe), 3.10 (6-OMe), 2.38/2.33 (NMe); MS (ESI, *m/z*): 1505.6 [M+H^+^], 1540.4 [M+Cl^−^]. Anal. Calcd. for C_77_H_136_N_2_O_26_: C, 61.41; H, 9.10; N, 1.86. Found: C, 61.43; H, 9.06; N, 1.85.

**1l**

White solid, yield 20%; ^13^C NMR (75 MHz, CDCl_3_): δ 222.0/172.6 (C-9), 175.7/175.0 (C-1), 102.9/102.7 (C-1'), 96.5/96.3 (C-1''), 84.7/84.4 (C-5), 80.5/80.2 (C-3), 77.9 (C-4''), 76.8 (C-13), 74.9/74.7 (C-6), 74.6/74.5 (C-12), 72.9/72.8 (C-3''), 70.9/70.8 (C-2'), 70.4/68.3 (C-11), 69.0/68.4 (C-5'), 65.7/64.4 (C-3'), 65.5 (C-5''), 56.2 (NCH_2_), 49.5 (3''-OMe), 45.1/26.9 (C-8), 44.9/44.8 (C-2), 39.3/38.8 (C-4), 38.6/37.8 (C-7), 37.5/33.0 (C-10), 37.4/36.4 (NMe), 35.1/35.0 (C-2''), 30.2/29.4 (C-4'), 26.8 (C-6 Me), 22.7 (C-3'' Me), 21.5 (C-5' Me), 21.1 (C-14), 18.7/18.5 (C-8 Me), 18.6/18.3 (C-5'' Me), 16.3/16.0 (C-2 Me), 16.2 (C-12 Me), 14.8/12.0 (C-10 Me), 10.7/10.6 (C-15), 9.5/9.4 (C-4 Me), 97.5, 71.9, 68.3, 59.1 (OCH_2_OCH_2_CH_2_OCH_3_), 130.9/ 128.8 (CH=CH); ^1^H NMR (300 MHz, CDCl_3_): δ 5.30 (CH=CH), 5.10/5.04 (H-13), 4.87 (H-1''), 4.44 (H-1'), 3.31 (3''-OMe), 2.52/ 2.46 (NMe), 5.18, 3.74, 3.57, 3.42 (OCH_2_OCH_2_CH_2_OCH_3_); MS (ESI, *m/z*): 1596.0 [M+H^+^], 798.4 [M/2+H^+^]. Anal. Calcd. for C_80_H_143_N_3_O_28_: C, 60.24; H, 9.04; N, 2.63. Found: C, 60.18; H, 9.07; N, 2.61.

**1m**

White solid, yield 19%; ^13^C NMR (75 MHz, CDCl_3_): δ 222.1/172.1 (C-9), 175.7/175.0 (C-1), 103.0/102.8 (C-1'), 96.4 (C-1''), 83.9 (C-5), 80.1/80.0 (C-3), 78.6/78.3 (C-4''), 76.2/76.1 (C-13), 76.0/74.9 (C-6), 74.6/74.2 (C-12),72.8/72.7 (C-3''), 70.9/70.7 (C-2'), 70.5/68.9 (C-11), 68.6/68.1 (C-5'), 65.6 (C-5''), 64.7/64.6 (C-3'), 55.9/55.8 (NCH_2_), 49.5/49.4 (3''-OMe), 45.1/44.6 (C-2), 44.8/26.7 (C-8), 39.3/39.0 (C-4), 38.7/37.6 (C-7), 37.8/33.0 (C-10), 37.3/36.4 (NMe), 35.0/34.9 (C-2''), 29.3/28.9 (C-4'), 26.9/26.5 (C-6 Me), 21.5 (C-3'' Me), 21.4 (C-5' Me), 21.2/21.0 (C-14), 18.6 (C-5'' Me), 18.5/18.2 (C-8 Me), 16.3/16.1 (C-12 Me), 16.0 (C-2 Me), 14.5/11.9 (C-10 Me), 10.9/10.6 (C-15), 9.3/9.2 (C-4 Me), 130.9/128.7 (CH=CH), 137.3, 128.5, 128.2, 128.0, 75.1 (OBenzyl); ^1^H NMR (300 MHz, CDCl_3_): δ 5.78 (CH=CH), 5.12 (H-13), 4.90 (H-1''), 4.42/4.39 (H-1'), 3.31 (3''-OMe), 2.38/2.33 (NMe), 7.70, 7.54, 7.34, 5.05 (OBenzyl); MS (ESI, *m/z*): 1597.5 [M+H^+^], 799.6 [M/2+H^+^], 1631.6 [M+Cl^−^]. Anal. Calcd. for C_83_H_141_N_3_O_26_: C, 62.42; H, 8.90; N, 2.63. Found: C, 62.37; H, 8.88; N, 2.61.

**1n**

White solid, yield 24%; ^13^C NMR (75 MHz, CDCl_3_): δ221.0/172.7 (C-9), 175.8/175.1 (C-1), 103.0/102.6 (C-1'), 96.1 (C-1''), 84.1/81.0 (C-5), 80.2/78.3 (C-3), 77.9 (C-4''), 77.0 (C-13), 78.4/74.7 (C-6), 74.2 (C-12), 72.7 (C-3''), 71.1/70.8 (C-2'), 70.4/69.0 (C-11), 68.6 (C-5'), 65.8/65.4 (C-5''), 64.8/64.6 (C-3'), 55.7 (NCH_2_), 50.6 (6-OMe), 49.5 (3''-OMe), 45.2/44.7 (C-2), 45.0/26.9 (C-8), 39.3/37.3 (C-7), 39.1/38.8 (C-4), 37.4/36.4 (NMe), 37.2/33.0 (C-10), 35.0/34.9 (C-2''), 30.3/29.3 (C-4'), 26.8/19.7 (C-6 Me), 21.5 (C-3'' Me), 21.4 (C-5' Me), 21.3/21.0 (C-14), 18.7/18.0 (C-8 Me), 18.5 (C-5'' Me), 16.2 (C-12 Me), 16.0 (C-2 Me), 14.8/12.3 (C-10 Me), 10.6 (C-15), 9.2/9.1 (C-4 Me), 97.4, 71.8, 68.3, 59.0 (OCH_2_OCH_2_CH_2_OCH_3_), 130.9/128.8 (CH=CH); ^1^H NMR (300 MHz, CDCl_3_): δ 5.68 (CH=CH), 5.12/5.06 (H-13), 4.92/4.87 (H-1''), 4.44/4.41 (H-1'), 3.32/3.31 (3''-OMe), 3.04 (6-OMe), 2.29 (NMe), 5.18, 3.75, 3.56, 3.42 (OCH_2_OCH_2_CH_2_OCH_3_); MS (ESI, *m/z*): 1609.4 [M+H^+^], 1644.4 [M+Cl^−^], 1654.2 [M+HCOO^−^]. Anal. Calcd. for C_81_H_145_N_3_O_28_: C, 60.46; H, 9.08; N, 2.61. Found: C, 60.43; H, 9.11; N, 2.60.

**2a**

White solid, yield 77%; ^13^C NMR (75 MHz, CDCl_3_): δ 175.1 (C-1), 148.6 (C-9), 102.7 (C-1'), 100.4 (C-8), 96.6 (C-1''), 87.0 (C-6), 81.2 (C-5), 79.6 (C-3), 77.1 (C-4''), 76.5 (C-11), 76.0 (C-13), 75.6 (C-12), 71.6 (C-3''), 69.7 (C-2'), 67.6 (C-5'), 64.3 (C-5''/C-3'), 57.4 (NCH_2_), 48.4 (3''-OMe), 45.8 (C-2), 42.3 (C-7), 37.7 (C-4), 35.4 (NMe), 34.2 (C-2''), 30.6 (C-10), 28.7 (C-4'), 25.7 (C-6 Me), 21.5 (C-14), 20.1 (C-5' Me), 20.5 (C-3'' Me), 17.4 (C-5'' Me), 15.4 (C-12 Me), 14.2 (C-2 Me), 11.0 (C-15), 10.3 (C-10 Me), 10.1 (C-8 Me), 8.6 (C-4 Me), 130.9 (CH=CH); ^1^H NMR (300 MHz, CDCl_3_): δ 5.84 (CH=CH), 5.05 (H-13), 4.89 (H-1''), 4.27 (H-1'), 3.28 (3''-OMe), 2.40 (NMe); MS (ESI, *m/z*): 1456.1 [M+H^+^], 1479.0 [M+Na^+^]. Anal. Calcd. for C_78_H_138_N_2_O_26_: C, 62.70; H, 9.00; N, 1.92; Found: C, 62.62; H, 9.04; N, 1.91.

**2b**

White solid, yield 61%; ^13^C NMR (75 MHz, CDCl_3_): δ 173.6 (C-1), 118.1 (C-9), 103.9 (C-1'), 98.8 (C-1''), 38.7 (C-8), 88.5 (C-12), 87.1 (C-11), 83.6 (C-6/C-5), 78.5 (C-3/C-4''), 77.0 (C-13), 72.8 (C-3''), 69.1 (C-2'), 68.1 (C-5'), 65.0 (C-5''/C-3'), 57.5 (NCH_2_), 49.2 (3''-OMe), 46.6 (C-2), 42.8 (C-7), 39.3 (C-4), 30.9 (C-10), 35.4 (NMe), 35.2 (C-2''), 30.3 (C-4'), 28.9 (C-6 Me), 24.0 (C-14), 20.6 (C-5' Me), 21.3 (C-3'' Me), 18.0 (C-5'' Me), 19.7 (C-12 Me), 15.0 (C-2 Me/C-8 Me), 11.7 (C-15), 15.3 (C-10 Me), 10.2 (C-4 Me), 130.9 (CH=CH); ^1^H NMR (300 MHz, CDCl_3_): δ 5.73 (CH=CH), 4.76 (H-1''), 4.23 (H-1'), 3.24 (3''-OMe), 2.38 (NMe); MS (ESI, *m/z*): 1456.1 [M+H^+^], 1478.1 [M+Na^+^]. Anal. Calcd. for C_78_H_138_N_2_O_26_: C, 62.70; H, 9.00; N, 1.92; Found: C, 62.73; H, 9.01; N, 1.87.

**Bioassay**

1. Reagent and Cell culture

AnnexinV-FITC/PI Assay Kit and Cell Mitochondria Isolation Kit were purchased from Beyotime Institute of Biotechnology, Jiangsu, China; DNA Ladder Detection Kit from Dingguochangsheng Biotechnology, Beijing, China; Mouse β-actin, Rabbit Bax, Rabbit Bcl-2, Rabbit caspase-3, Rabbit Cytochrome-C were purchased from Proteintech, Wuhan, China.

All cell lines were purchased from American Type Culture Collection (ATCC, Manassas, VA, USA). Non-small cell lung carcinoma cells (A549), hepatocellular carcinoma cells (HepG-2) and breast carcinoma cells (MCF-7) were cultured in DMEM medium (Sigma, St. Louis, MO, USA), while gastric carcinoma cells (BGC-823), laryngeal carcinoma cells (Hep2), cervical carcinoma cells (HeLa) were cultured in RPMI-1640 medium (Sigma, St. Louis, MO, USA), which were supplemented with 10% fetal bovine serum (FBS; TBD, Tianjin, China) at 37℃ in humidified atmosphere with 5% CO_2_.

1. MTT assay

The antiproliferative activity of the synthetic compounds was measured by 3-(4, 5- dimethylthiazol-2-yl)-2, 5-diphenyltetrazolium bromide (MTT) assay. Cells were seeded in 96-well plates (Corning, NY, USA) at a density of 8×10^3^ per well. After 24 h of incubation, cells were treated with different concentrations (0.1, 0.3, 1, 3 and 10 µg/ml) of the EM-A dimers for the indicated time periods. Afterwards, MTT (Sigma, St. Louis, MO) solution [5.0 mg/ml in phosphate-buffered saline (PBS)] was added (20 µl/well) and incubated for another 4 h at 37℃. The purple formazan crystals were then dissolved in 100 µl dimethyl sulfoxide (DMSO). After 5 min, the plates were read on a plate microreader (TECANSPECTRA, Wetzlar, Germanay) at 490 nm. The IC_50_ values were obtained using the software of Dose–Effect Analysis with Microcomputers and were deﬁned as concentration of drug causing 50% inhibition in absorbance compared with control cells. Assays were performed in triplicate from three independent experiments.

1. Cell cycle analysis

Cells were dispensed in 25 ml culture bottle at a density of 5 ×10^5^ per bottle. After 24 h incubation, they were treated with compound **1b** at given concentrations for 24h. The cells were harvested by 0.05% trypsin (Sigma, St. Louis, MO), then collected by centrifugation, washed with PBS, and ﬁxed with 10 ml ice-cold 70% ethanol at 4°C overnight. After washed with PBS, the cells were suspended in 1ml propidium iodide (PI; 50 mg/L; Sigma, St. Louis, MO) solution supplemented with 1 g/L RNase A (Sigma, St. Louis, MO). Finally, the samples were analyzed by FACScan flow cytometer (Becton Dickinson, Franklin, NJ, USA).

1. Cell morphology observation and DNA fragmentation

Cells were seeded in 6-well plates at a density of 1.2×10^5^ (MCF-7) or 1.5×10^5^ (HeLa) per well. After 24 h of incubation, cells were treated with 0 or 0.15 μM **1b** for another 24h and then observed under a phase contrast microscope.

Cells were seeded in 6-well plates at a density of 0.8×10^5^ (MCF-7) or 1.0×10^5^ (HeLa) per well. After 24 h of incubation, cells were treated with 0, 0.5, 1.5 or 2.5 μM **1b** for another 24h. Subsequently, cells were washed with PBS twice, stained with AO-EB working solution, and observed under a fluorescent microscope (Olympus, Tokyo, Japan).

Cells were seeded in 6-well plates at a density of 1.2×10^5^ (MCF-7) or 1.5×10^5^ (HeLa) per well. After 24 h of incubation, cells were treated with 0, 0.5, 1.5 or 2.5 μM **1b** for another 24h. DNA ladder detection kit was used to extract the DNA, and 30 μL of DNA sample was loaded onto a 0.8 % agarose gel which was run at 5 V/cm for 1.5 h. The stained DNA was observed by transillumination with UV light and photographed.

1. Annexin V-FITC apoptosis analysis

Cell apoptosis was determined using AnnexinV-FITC/PI Assay Kit according to the manufacturers’ protocols. Briefly, cells were seeded in 6-well plates at a density of 1.2×10^5^ (MCF-7) or 1.5×10^5^ (HeLa) per well and incubated for 24 h. Then, cells were treated with 0, 0.5, 1.5 or 2.5 μM **1b** for 24h. After washed with PBS twice, cells were re-suspended in 100 μL binding buffer containing 5 μL AnnexinV-FITC and 5 μL PI. Following 10 min of incubation, cells were detected by FACScan flow cytometer.

1. Mitochondrial potential assay

Cells were seeded in 6-well plates at a density of 0.8×10^5^ (MCF-7) or 1.0×10^5^ (HeLa) per well. After 24 h of incubation, cells were treated with 0, 0.5, 1.5 or 2.5 μM **1b** for another 24h. Subsequently, cells were washed with PBS twice, incubated with 1 μg/mL Rhodamine 123 in 1 mL PBS at 37°C for 30 min, and analyzed by FACScan flow cytometer.

1. Western blot analysis

Cells were seeded in 75 mL culture flask at a density of 8 ×10^5^ (MCF-7) or 1.2×10^6^ (HeLa) and incubated for 24 h. Thereafter, cells were treated with 0, 0.5, 1.5 or 2.5 μM **1b** for 24h and harvested by trypsin into the EP tubes.

For total protein extraction, the cell pellets were lysed in RIPA buffer (Beyotime) supplemented with PMSF on ice for 30 min. After centrifuging at 12000 g for 10 min at 4℃, the cell suspension was collected as the whole cell protein. Cytoplasmic proteins and mitochondrial proteins were extracted from cells using Cell Mitochondria Isolation Kit according to the instructions of manufacturer. The protein concentrations were quantified with BCA Protein Assay Kit (Beyotime) and a plate reader according to the manufacturer’s protocols.

For western blot analysis, equal protein lysates were separated by electrophoresis on 12% SDS-PAGE gels, and transferred onto PVDF membranes. After blocking with 5% non-fat milk in PBS for 2.5 h at room temperature, the membranes were incubated with primary antibody overnight at 4℃: Bax (1:2000), Bcl-2 (1:1000), Caspase-3 (1:500), Cytochrome c (1:500), and β-actin (1:3000). Then, the blots were washed three times for 10 min each in Tris-NaCl and incubated with secondary HRP-conjugated goat anti-mouse or anti-rabbit IgGs (1:6000 or 1:5000) for 2.5 h at 37℃. The interest proteins were visualized using ECL, and β-actin served as the internal control.

1. Statistical analysis

Data were expressed as mean ± SD (standard deviation) from three independent experiments. Student’s *t* tests were used to compare the means of two groups. p < 0.05 was considered as statistical significance.
